# Study-test overlap rather than multisensory integration benefits memory

**DOI:** 10.3758/s13421-025-01759-0

**Published:** 2025-07-21

**Authors:** Diane Pecher, Brandon Keytel, René Zeelenberg

**Affiliations:** Department of Psychology, Child and Educational Studies, Erasmus University Rotterdam, room T16-39, Postbus 1738, 3000 DR, Rotterdam, The Netherlands

**Keywords:** Multisensory memory, Multisensory integration, Encoding specificity, Continuous recognition

## Abstract

Previous research has obtained better memory performance for items that were studied in two modalities than in one modality. We investigated the role of multisensory integration and of study-test overlap. Items were presented once as unimodal (picture or sound), once as bimodal (picture and sound), or twice as unimodal (once as picture, once as sound) stimuli in a continuous recognition task. In Experiment 1 we found a benefit for items that were studied in both modalities. The benefit did not depend on temporal alignment of picture and sound, which poses problems for multisensory integration as an explanation. In Experiment 2 we found that repetition of items in the same modality resulted in better memory performance than repetition in different modalities, and we found that memory performance was better when study and test format were the same than when they were different, supporting a role for encoding specificity. We conclude that multimodal presentation during study benefits memory only when the test item is also multimodal. Moreover, this benefit is more likely explained by study-test overlap than by multisensory integration.

## Introduction

Memory performance benefits from overlap in the presentation format or type of processing between study and test (e.g., Blaxton, 1989; Cabeza, 1994; Parks, 2013; Roediger & Blaxton, 1987, Zeelenberg, 2005; see also Roediger et al., 1989), formulated as the encoding specificity principle (Tulving & Thomson, 1973) and the principle of transfer-appropriate processing (Morris et al., 1977). An apparent exception (Matusz et al., 2017; Meyerhoff et al., 2022) is the finding that events that are experienced in multiple modalities are better remembered than events that are experienced in a single modality even when the test is in a single modality (Duarte et al., 2023; Heikkilä et al., 2015; Lehmann & Murray, 2005; Moran et al., 2013; Thelen et al., 2015). For example, recognition memory for a picture of a car might be better if the picture had been studied with a congruent sound (i.e., that of a car engine) than if the picture had been studied alone (i.e., without a sound). A different prediction is made by the encoding specificity account. Because processing a picture will differ depending on the presence and congruency of a concurrent sound, the encoding specificity account predicts a memory benefit for pictures that were studied without a sound if the test also consists of pictures presented without sound.

The benefit for multimodal study items over unimodal study items has been explained by multisensory integrative processing at study (Quak et al., 2015; Shams & Seitz, 2008). The idea is that multisensory processing involves several modality-specific brain areas and that integrating those processing areas results in a stronger memory trace than when only one modality is involved. Studies have shown that multisensory processing activates additional brain areas that are not involved in unisensory processing (Amedi et al., 2005; Scheliga et al., 2023) and primary sensory cortical areas are also involved in multisensory processing (Ghanzanfar & Schroeder, 2006; Murray et al., 2016). Further evidence for the multisensory integration account is that the memory benefit depends on semantic congruency of the picture and sound (e.g., a picture of a dog and the sound of a barking dog; Heikkila et al., 2015, 2017b; Lehmann & Murray, 2005; Moran et al., 2013), which suggests it is not merely the presence of a second modality (e.g., a picture of a dog and the sound of a train) that causes the memory benefit, although Marian et al. (2021) did find a memory benefit for pictures that were presented with incongruent sounds. Overall, however, memory is expected to be better for congruent multimodal study items than for unimodal study items because a stronger, integrated representation is encoded for the target.

The evidence for a benefit of multisensory study, however, is mixed. Although some studies that presented unimodal items at test reported better memory for items that were studied multimodally than unimodally (Duarte et al., 2023; Lehmann & Murray, 2005; Moran et al., 2013; Thelen et al., 2015), not all studies have obtained such a benefit (Cohen et al., 2009; Nyberg et al., 2000; Pecher & Zeelenberg, 2022). Thus, the question remains if multisensory study benefits memory compared to unisensory study.

One potential explanation for the mixed results might lie in the differences between studies in the task-relevance of the different modalities. In several studies researchers have tested memory in the continuous recognition paradigm in which participants see a stream of items and make old/new recognition responses to each item. Typically, only one modality is task-relevant, for example, participants see a stream of pictures to which they respond and only a subset of these pictures is accompanied by sounds, which are task-irrelevant. In such cases, the effect of the additional modality might be minimal, because for multisensory integration processes attention to both modalities might be required (Doehrmann & Naumer, 2008; Quak et al., 2015). Thus, the differences in findings might be caused by differences in relevance of the additional modality. In Thelen et al., (2015; see also Heikkila et al., 2015, 2017b) the same participants performed a picture-recognition task and a sound-recognition task. They did so in different sessions but using the same stimuli in the picture and sound recognition task. In contrast, in Pecher and Zeelenberg (2022) participants performed only one recognition task so that during the entire experiment only one modality was task-relevant. Thus, even though in most studies only one modality was task-relevant at any given moment, participants who performed different recognition tasks might still have paid more attention to the irrelevant modality because the stimuli that were presented in the task-irrelevant modality for the current task were presented in the task-relevant modality in the other task, despite instructions to pay attention to one modality only.

In the experiments that follow we presented a memory task in which participants had to respond to both pictures and sounds. To increase our chances of finding a multisensory memory benefit, we modified the task so that pictures and sounds were task-relevant on each trial. We used the same continuous recognition task as in the previous experiments but now participants responded on whether a stimulus, either a sound or a picture, was presented for the first time (new) or the second time (old). For bimodal stimuli they responded “old” if either sound or picture (or both) were repeated. Thus, participants had to pay attention to both modalities.

In the present study we addressed the roles of study-test overlap and multisensory integration in the multisensory memory benefit. As mentioned above, the benefit of overlap in the presentation format of an item between study and test has been well established. Therefore, it is surprising that the multisensory study benefit is found when items are tested unimodally. Two recent studies, however, have found little or no multisensory study benefit for unimodally tested items, but instead found effects of study-test overlap (Meyerhoff et al., 2022; Pecher & Zeelenberg, 2022). In the present study, we presented items unimodally and multimodally at both study and test to assess the effect of study-test overlap. Based on the encoding specificity and transfer-appropriate processing principles, one might expect better performance if the format of items were the same during study and test, compared to when they were different.

Multisensory integration (Shams & Seitz, 2008) has been proposed as an explanation for the multisensory memory benefit by several authors (Duarte et al., 2023; Lehmann & Murray, 2005; Moran et al., 2013; Thelen et al., 2015). An alternative explanation for the multisensory study advantage, however, is the storage of more information rather than the integration of information from different modalities (Broadbent et al., 2020; Guazzo et al., 2020; Heikkila et al., 2017a, 2017b; Hendriks et al., 2024; Meyerhoff et al., 2022; Pecher & Zeelenberg, 2022; Thompson & Paivio, 1994). In the experiments that found a multisensory memory advantage for object pictures or sounds more information was presented for items studied in multimodal format than for items studied in unimodal format. For example, on a trial that consisted of a picture of a dog accompanied by the sound of a barking dog, more information related to the target object dog was presented than on trials with only the picture of a dog. Note that this issue is not addressed by multisensory incongruent trials, such as a picture of a dog with the sound of a hammer. Even though more information is provided on incongruent trials than on unisensory trials, the information is about different items. In the present study we tested the idea that more information about the same item might increase memory performance for that item. It might be simply the difference in amount of information rather than multisensory integration that explains the advantage for multimodal over unimodal items.

Multisensory integration in picture-sound experiments should depend on spatial and temporal alignment (Evans, 2020; Talsma et al., 2010). The hypothesized benefit of multisensory integration is that not only the picture and sound are processed in their modality-specific processing systems, but that additional processing occurs in multisensory or supramodal processing systems, resulting in integrated memory traces (Shams & Seitz, 2008). This additional processing requires that picture and sound are processed on the same trial. Some results indicate that the multisensory memory benefit might disappear when the amount of information is controlled. In a study in which children implicitly learned to categorize frogs, Broadbent et al., (2020; see also Heikkilä et al., 2017a) showed that categorization performance was affected by the amount of information that was presented about items during study but not by whether the information was in the same modality (e.g., two visual features) or different modalities (e.g., a visual and an auditory feature). A similar result was obtained by Guazzo et al. (2020), who found no difference between unimodal and crossmodal feature binding in a working memory task. Thus, multimodal study items may result in better memory than unimodal study items because more information about the item is presented during study rather than because information from different modalities has been integrated.

To disentangle the effects of multisensory integration and the amount of information presented, we presented study items as a simultaneous presentation of the picture and sound (bimodal condition) or as separate presentations of the picture and sound (unimodal-both condition) with several intervening unrelated items. Meyerhoff and Huff (2016) presented items in the auditory and visual modality sequentially (without intervening items), which may have resulted in some degree of multisensory integration. With intervening items, however, multisensory integration of auditory and visual items presented on separate trials would be much less likely (Meyerhoff & Huff, 2016; Meyerhoff et al., 2022). In Experiment 1 we tested whether any memory advantage for bimodal items is due to multisensory integration at encoding or could be explained simply by learning more information about an item. If integration is crucial, memory should be better for items when picture and sound are studied simultaneously than when picture and sound are studied on separate trials. If instead the amount of information is crucial, there should be no effect of temporal overlap, but memory should be better if both picture and sound are studied than if only one modality is studied. To be clear, our experiments are not aimed at testing if multisensory integration occurs but rather if it results in a memory benefit. We used the continuous recognition paradigm as in previous studies of the multisensory memory benefit (Lehmann & Murray, 2005; Moran et al., 2013; Pecher & Zeelenberg, 2022; Thelen et al., 2015). In a continuous recognition paradigm (Shepard & Teghtsoonian, 1961) the participants distinguish study (initial presentation) and test (repeated presentation) items in the same continuous stream of items. Unlike Thelen et al., who presented items several times in different blocks, we presented each item only once for study and once for test.

## Experiment 1

### Method

#### Participants

Ninety-three students at the Erasmus University participated for course credit. This sample size provided a power of 0.95 to detect a small effect (*f* = 0.15) in the repeated-measures ANOVA.

#### Stimuli

A set of 138 picture-sound pairs was used as congruent pairs. Of these, 130 were used in the experimental trials and eight were used for practice. The pictures were colored line drawings (281 by 197 pixels) of animals (e.g., *dog*, *bee*) and objects (e.g., *trumpet*, *helicopter*). The pictures were selected from the stimuli used by Pecher and Zeelenberg (2022) and Moran et al. (2013), kindly provided by Zachary Moran. The sound files were 1,000-ms clips of a typical sound made by the animal or object. They were retrieved from various websites with free sound clips and approximately matched in loudness. We selected longer clips than those used by previous studies to improve the identifiability of the sounds. A full list of items is provided in Appendix A. For each participant, a different random allocation of items to conditions was created.

#### Design

Three study conditions were used: unimodal, unimodal-both, and bimodal. In the unimodal study condition, an item was presented once in one modality, either as a picture or as a sound. In the unimodal-both study condition, the item was presented twice, once as a picture and once as a sound, on different trials separated by one to eight intervening items. For half of the items the sound was presented first, for the other half the picture was presented first. Finally, in the bimodal study condition the item was presented as a picture and a sound simultaneously. Thus, in the unimodal-both and the bimodal condition the same information was studied; the only difference was whether the picture and sound were presented simultaneously or on separate trials. The test conditions were unimodal or bimodal. The study and test conditions were fully crossed, except for items in the unimodal study – unimodal test condition which were always presented in the same modality at study and test. The number of test items for each type of trial are presented in Table 1.

**Table 1:** Presentation conditions and number of items in each test condition for Experiment 1

Study	Test		
	Unimodal picture	Unimodal sound	Bimodal
Unimodal-picture	13		13
Unimodal-sound		13	13
Unimodal-both	13	13	13
Bimodal congruent	13	13	13

#### Procedure

**Fig. 1:**
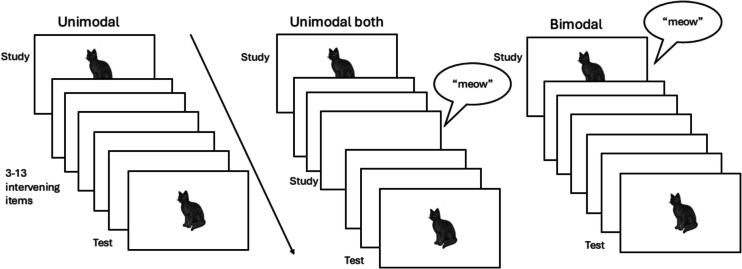
Timeline of the procedure in three unimodal test conditions. Figure 1 illustrates the three study conditions for the unimodal visual test condition. In the unimodal study condition, a picture was presented on the study trial. In the unimodal-both condition, a picture and a sound were presented on two separate study trials. In the bimodal condition, the picture and sound were presented simultaneously on the study trial. Note that the experiment included additional test conditions (unimodal sound and bimodal) which are not shown in the figure

The experimental procedure was closely based on that of Pecher and Zeelenberg (2022) and Thelen et al. (2015). Participants were tested individually. They were seated at a desk wearing headphones with a keyboard and monitor on the desk in front of them, all connected to the PC that was used to run the experiment. Before the start of the experiment a few filler sound files were played so that participants could adjust the volume to a comfortable level. Participants were instructed that a list of pictures and sounds would be presented and they were to decide for each picture and sound whether it was “old” or “new.” The instructions indicated that they should respond to specific items, thus in the unimodal-both study condition participants should respond “new” to both the picture and the sound, because each was presented for the first time. If a bimodal item was presented, they should respond “old” if either picture or sound (or both) had been presented before. The experiment started with 20 practice trials, followed by 299 experimental trials. Each trial started with a 500-ms fixation marker (+) in the center of the screen, followed by a stimulus that was presented for 1,000 ms. Each stimulus was followed by a variable inter-stimulus interval (ISI) of 2,900–3,500 ms^1^ until the next trial started. Participants kept their index fingers on the Z and M keys throughout the block. They pressed the Z key to indicate an item was new and the M key to indicate an item was old. After each practice trial, the word “Correct” in blue or “Incorrect” in red was displayed in the center of the screen for 250 ms. During the experimental trials no feedback was provided. Items from different conditions were mixed in a semi-random order. Repetitions were presented at a lag that varied from three to 13 items. The average lag between the two study trials in the unimodal-both condition was 4.1. The three study conditions are illustrated in Fig. 1.

**Fig. 2A:**
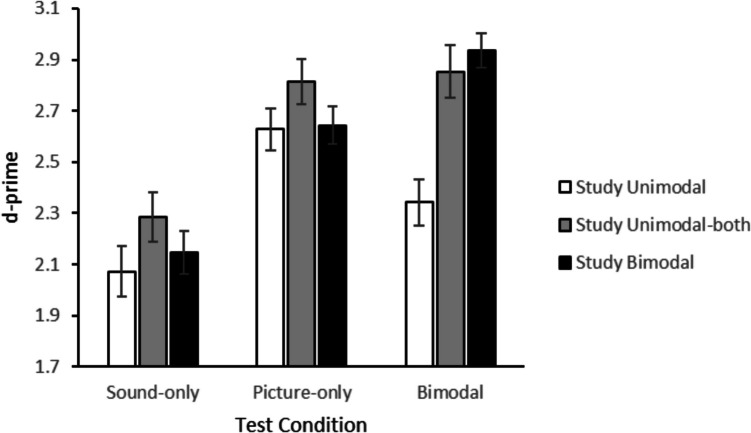
verage d-primes in Experiment 1 (error bars represent standard errors)

### Results

Data from 11 participants were excluded because their accuracy was below 60% (as was done in Pecher & Zeelenberg, 2022), leaving data for 82 participants for the analyses. Only trials on which a participant responded were included (95.8% of all trials). All data described in this section are available via the Open Science Framework (OSF) at: https://osf.io/9m2jv/. The accuracy for new and old items was calculated for each condition. The mean hit and false alarm rates for the three study conditions are presented in Table 2. The mean proportion of correct responses to study items (i.e., correct rejections) are shown in Table 3. False alarms were calculated as the proportion of incorrect “old” responses to study items of the same format. For each type of trial *d’* (d-prime) was calculated using the Snodgrass and Corwin (1988) correction to prevent hit and false alarms values of 1 and 0. The hit rate was calculated as $H=\frac{\#hits+0.5}{\#old trials+1}$. The false alarm rate was calculated as$FA=\frac{\#false alarms+0.5}{\#new trials+1}$. The values for *d’* were calculated as *d’* = *z(H)—z(FA)*. Because hit rates (and false alarms) are influenced by response bias, *d’* provides a better measure of memory performance than hit rates. The false alarm rates were calculated from “old” responses to the first presented items grouped by study condition. The average *d’*s are shown in Fig. 2.

**Table 2:** Mean hit rates and false alarm rates (with standard error of the mean) in Experiment 1

Test	Study	Hit rates		False alarms	
Sound only	Unimodal-sound	0.803	(0.019)	0.166	(0.013)
	Unimodal-both	0.859	(0.011)		
	Bimodal	0.817	(0.018)		
Picture only	Unimodal-picture	0.845	(0.020)	0.104	(0.010)
	Unimodal-both	0.890	(0.013)		
	Bimodal	0.845	(0.019)		
Bimodal	Unimodal-sound	0.693	(0.024)	0.097	(0.008)
	Unimodal-picture	0.831	(0.020)		
	Unimodal-both	0.900	(0.010)		
	Bimodal	0.918	(0.007)		

False alarm rates (“old” responses to first presentations) are given with the test format that corresponds to the format of the first presentation. Hit and false alarm rates are values obtained after applying the Snodgrass and Corwin (1988) correction. In the bimodal test condition, the unimodal-sound and unimodal-picture condition were combined into one unimodal condition, as also shown in Fig. 2

**Table 3:** Mean correct responses to study items (with standard error of the mean) in Experiment 1

	Presentation 1		Presentation 2	
Unimodal-picture	0.819	(0.028)	0.721	(0.030)
Unimodal-sound	0.758	(0.026)	0.692	(0.031)
Bimodal	0.807	(0.026)		

For the overall analysis we combined the responses to bimodally tested items that were studied unimodally as a sound and those studied unimodally as a picture into a single unimodal condition. A 3 (study condition) × 3 (test condition) ANOVA on the *d’* values showed that memory was better for items studied in two modalities (unimodal-both *M* = 2.65 and bimodal *M* = 2.57) than for items studied in one modality (*M* = 2.35), *F*(2, 162) = 43.88, *p* < 0.001, partial *η*^2^ = 0.35. Test format also had an effect on memory performance, memory was better for items tested in the bimodal (*M* = 2.71) or a picture-only (*M* = 2.70) condition than for items tested in the sound-only (*M* = 2.17) condition, *F*(2, 162) = 32.80, *p* < 0.001, partial *η*^2^ = 0.29. Finally, the interaction was significant, *F*(4, 324) = 11.19, *p* < 0.001, partial *η*^2^ = 0.12. As can be seen in Fig. 2, the effect of study condition was different for the three test conditions. Follow-up analyses were performed to gain more insight in the results.

To test whether temporal overlap between sound and picture affected memory we performed a follow-up 2 (study: unimodal-both vs. bimodal) × 3 (test: sound only, picture only, or bimodal) ANOVA so that we could directly compare the study condition with presentation of sound and picture on different trials (unimodal-both) to the study condition with simultaneous presentation (bimodal). This analysis showed better memory performance for items studied twice unimodally than items studied once bimodally, *F*(1, 81) = 5.39, *p* = 0.023, partial *η*^2^ = 0.06, and an interaction with test format, *F*(2, 162) = 5.50, *p* = 0.005, partial *η*^2^ = 0.06. Follow-up (uncorrected) *t*-tests showed that performance was better for items studied twice unimodally than once bimodally both when the test was sound only, *t*(81) = 2.06, *p* = 0.043, *BF*_*01*_ = 1.11,^2^ and when the test was picture only, *t*(81) = 3.09, *p* = 0.003, *BF*_*10*_ = 9.71. There was, however, no significant difference between items studied twice unimodally and once bimodally for items that were tested bimodally, *t*(81) = 1.63, *p* = 0.106, *BF*_*01*_ = 2.30.

We also performed follow-up analyses to test whether items that were tested unimodally benefited from bimodal study compared to unimodal study, that is, the multisensory benefit found in some previous studies. For items tested as sound only, memory performance did not differ between items studied as sound only and items studied bimodally, *t*(81) = 1.31, *p* = 0.195, *BF*_*01*_ = 3.62. Likewise, for items tested as picture only, memory performance did not differ between items studied as picture only and items studied bimodally, *t*(81) = 0.28, *p* = 0.780, *BF*_*01*_ = 7.90. These results replicate the findings of Pecher and Zeelenberg (2022), who did not find a benefit for bimodal study items relative to unimodal study items.

Next, we examined whether items benefitted from being studied in the unimodal-both condition. For items tested as sound only, memory was better for items studied twice unimodally than for items studied as sound only, *t*(81) = 2.91, *p* = 0.005, *BF*_*10*_ = 5.96. For items tested as picture only, memory was better for items studied twice unimodally than for items studied as picture only, *t*(81) = 2.80, *p* = 0.006, *BF*_*10*_ = 4.54.

To summarize, items tested as sound only or picture only benefited from being studied in the unimodal-both condition but did not benefit from bimodal study.

## Experiment 2

In Experiment 1 we found that for items that were tested unimodally, memory performance was enhanced for items studied in both the visual and the auditory modality, but only when picture and sound were presented on separate trials. That is, for items tested unimodally, there was no benefit of (single trial) bimodal study. This suggests that the benefit for the unimodal-both condition was due to item repetition rather than the presentation in two modalities. This benefit may be due to the additional presentation or total processing time for a concept (Meyerhoff et al., 2022) or repeated activation of the verbal label (Thelen et al., 2015). A crucial assumption here is that participants activate the same semantic concept during perception of the picture and sound (Marian et al., 2021). Memory performance will thus be better for items that are repeated than for items that are presented only once. To explore the role of item repetition, we presented all items twice for study. If the unimodal-both advantage was due to repetition, we expected that the unimodal-both advantage would disappear if all study conditions involved two presentations. Instead, we expected that performance would be best in those conditions in which there was most overlap between study and test. Thus, for unimodal test items performance would be best for items that were studied twice in the same unimodal condition, and for bimodal test items performance would be best for items that were studied twice in the bimodal condition.

### Method

#### Participants

One hundred and thirty-three students at Erasmus University participated for course credit. Of these, 128 participants (111 female, 17 male) were included. This sample size provided a power of 0.99 to detect a small effect (*f* = 0.15) in the repeated-measures ANOVA. Their average age was 20.4 (range 18–30) years. Five participants were excluded because their accuracy was lower than 60% (four) or they did not follow instructions (one).

#### Stimuli

A set of 204 picture-sound pairs was used, consisting of the items from Experiment 1 plus 66 additional items from the same sources. Four pairs were used for practice trials. Stimuli were randomly assigned to experimental conditions with a new random assignment for each participant.

#### Design

The three study conditions (unimodal, unimodal-both, and bimodal) were fully crossed with the three test conditions (sound only, picture only, bimodal). In the unimodal study condition an item was presented twice in the same modality (picture or sound). In the unimodal-both study condition, an item was presented once in one modality and once in the other modality. Half the items in the unimodal-both condition were presented as picture first and a sound second; the other half as sound first and picture second. In the bimodal study condition picture and sound were presented twice simultaneously. Thus, all items were presented twice during study. In the unimodal and bimodal conditions, the second study presentation was identical to the first, and thus participants should respond “old.” In the unimodal-both study condition the two study presentations were not identical, and thus participants should respond “new” on both trials. Repetitions were separated by one to seven intervening items. The average lag was 5.3 items. To prevent ceiling effects, we increased the study-test lag to 12–50 intervening items. The average lag was 40.5 items.

#### Procedure

The procedure was the same as in Experiment 1. The stimuli were presented in three blocks of 200 trials.

## Results

**Fig. 3:**
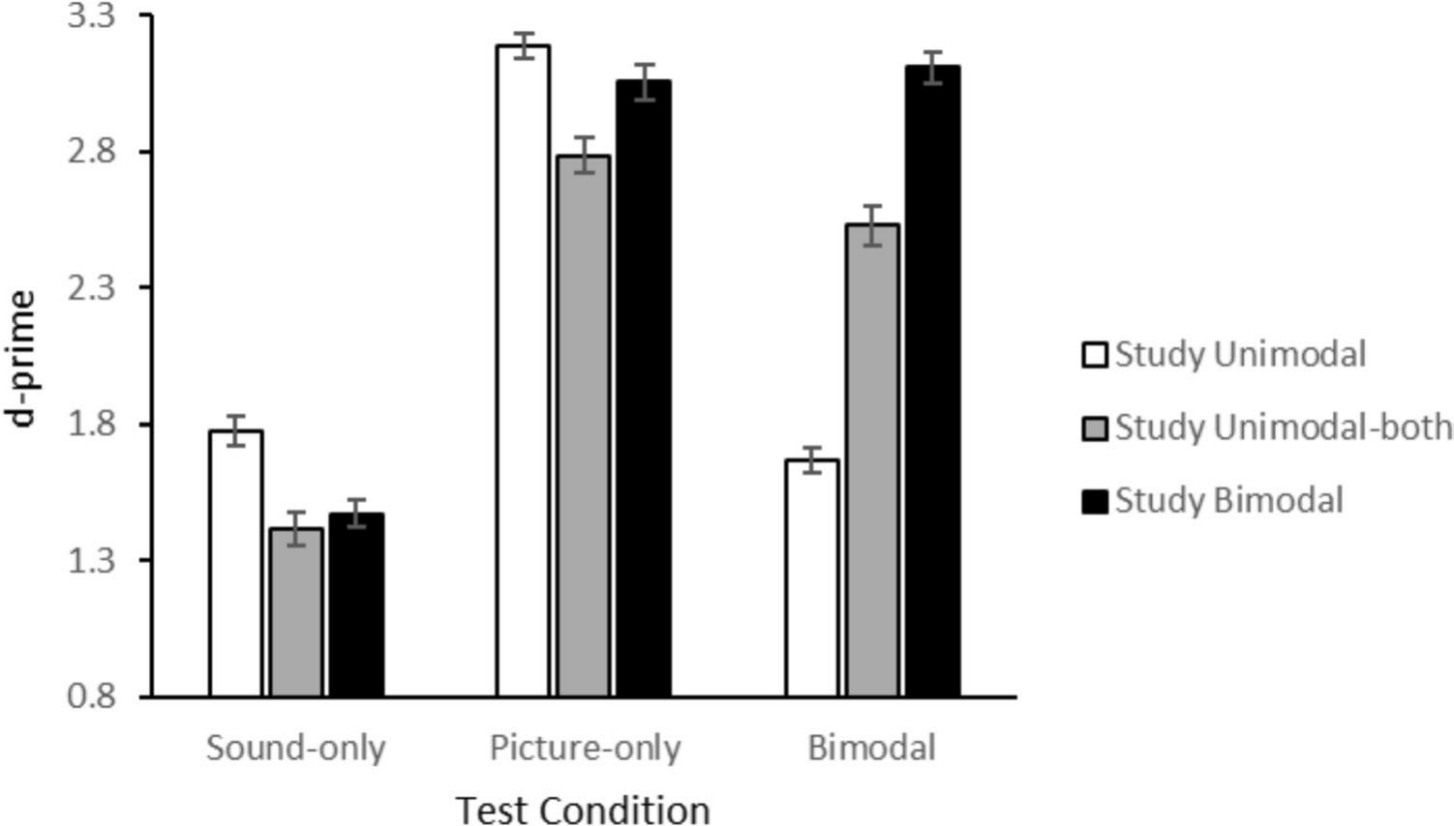
Average d-primes in Experiment 2 (error bars represent standard errors)

Only trials on which a participant responded were included (96.4% of all trials). All data described in this section are available via the OSF at: https://osf.io/9m2jv/. The accuracy for new and old items was calculated for each condition. The mean hit rates and false alarm rates for the three study conditions are presented in Table 4.. The mean proportion of correct responses to study items (i.e., correct rejections) is shown in Table 5. The average *d’s* for all conditions are shown in Fig. 3.

**Table 4:** Mean hit rates and false alarm rates to items tested as sound-only and picture-only items in Experiment 2

Test	Study	Hit rates		False alarms	
Sound only	Unimodal-sound	0.831	(0.012)	0.266	(0.011)
	Unimodal-both	0.745	(0.014)		
	Bimodal	0.759	(0.014)		
Picture only	Unimodal-picture	0.936	(0.006)	0.082	(0.009)
	Unimodal-both	0.866	(0.009)		
	Bimodal	0.903	(0.012)		
Bimodal	Unimodal-sound	0.418	(0.021)	0.117	(0.009)
	Unimodal-picture	0.806	(0.020)		
	Unimodal-both	0.833	(0.016)		
	Bimodal	0.951	(0.004)		

F﻿alse alarm rates (“old” responses to first presentations) are given with the test format that corresponds to the format of the first presentation. Hit and false alarm rates are values obtained after applying the Snodgrass and Corwin (1988) correction. In the bimodal test condition, the unimodal-sound and unimodal-picture condition were combined into one unimodal condition, as also shown in Fig. 3

**Table 5:** Mean correct responses to study items (with standard error of the mean) in Experiment 2

	Presentation 1		Presentation 2	
Unimodal-picture	0.888	(0.008)	0.879	(0.008)
Unimodal-sound	0.704	(0.012)	0.760	(0.009)
Bimodal	0.863	(0.009)	0.934	(0.006)

A 3 (study format) × 3 (test format) ANOVA showed an effect of test format, *F*(2,508) = 452.98, *p* < 0.001, partial *η*^2^ = 0.78. Memory performance was best in the picture-only test (*M* = 3.01), then in the bimodal test (*M* = 2.43) and worst in the sound-only test (*M* = 1.56). There was also an effect of study format, *F*(2,508) = 104.02, *p* < 0.001, partial *η*^2^ = 0.45, with better performance in the bimodal study condition (*M* = 2.55) than in the unimodal-both (*M* = 2.24) and unimodal (*M* = 2.21) study conditions. Finally, there was an interaction between study format and test format, *F*(4,508) = 202.59, *p* < 0.001, partial *η*^2^ = 0.62.

We subsequently compared performance in exactly overlapping study-test conditions to performance in the unimodal-both study condition for each test condition. For items tested as sound only, memory was better after unimodal-sound study than after unimodal-both study, *t*(127) = 8.76, *p* < 0.001, *BF*_*10*_ = 6.19 × 10^11^. Likewise, for items tested as picture only, memory was better after unimodal-picture study than after unimodal-both study, *t*(127) = 11.40, *p* < 0.001, *BF*_*10*_ = 1.27 × 10^18^. Finally, for items tested in the bimodal condition, memory was better after bimodal study than after unimodal-both study, *t*(127) = 9.81, *p* < 0.001, *BF*_*10*_ = 1.88 × 10^14^. Thus, for all three test conditions (i.e., sound only, picture only, and bimodal) performance was better when the study and test conditions overlapped than when items were studied in the unimodal-both condition.

We also performed follow-up analyses to test whether items that were tested unimodally benefited from bimodal study. For items tested as sound only, memory performance was better for items studied as sound only than for items studied bimodally, *t*(127) = 6.46, *p* < 0.001, *BF*_*10*_ = 4.87 × 10^6^. Likewise, for items tested as picture-only, performance was better for items studied as picture only than for items studied bimodally, *t*(127) = 2.85, *p* = 0.005, *BF*_*10*_ = 4.65. Thus, again we did not find a multisensory benefit for items that were tested unimodally. Rather, a multisensory deficit was found.

Finally, performance in the sound-only test did not differ between bimodal and unimodal-both study, *t*(127) = 1.18, *p* = 0.239, *BF*_*01*_ = 5.26. Performance in the picture-only test, however, was better after bimodal study than unimodal-both, *t*(127) = 5.14, *p* < 0.001, *BF*_*10*_ = 12.71 × 10^3^.

Thus, the results of Experiment 2 showed that study-test overlap has a positive effect on memory performance. In the unimodal test conditions, performance was better for items that had been studied twice in the same unimodal format. If one of the study presentations was in the other modality (as in the unimodal-both study condition), performance was worse. Also in the unimodal test conditions, performance was better after bimodal study than unimodal-both, but not better than unimodal, indicating that repeating the test modality was driving the advantage, rather than the addition of the other modality. For items tested in bimodal format, performance was best when they were studied as bimodal items, so here too the condition with greatest study-test overlap resulted in the best memory performance.

## General discussion

In two experiments we investigated if memory was better for test items studied as multimodal items than for items studied as unimodal items. In Experiment 1 we found that memory performance was better for items that were studied in two modalities. However, this benefit for studying an item in two modalities was restricted to the situation where the two modalities were presented on separate study trials. No benefit was obtained when the two modalities were presented simultaneously during study. Although the bimodal study condition was designed to promote multisensory integration, it did not lead to a memory benefit. Rather, the results indicated that memory performance benefited from item repetition. In Experiment 2, we controlled for differences in item repetition by repeated study presentations for items in all conditions. Here we found a study-test overlap effect; memory performance was better when items were presented in the same format during study and test than when they were presented in a different format during study and test. For unimodally tested items, there was no benefit of also having studied the item in another modality. In fact, for items that were tested unimodally performance was better if the item had been studied twice in the same modality than if it had been studied twice in different modalities. Moreover, simultaneous study presentations of items in two modalities impaired, rather than benefitted, performance for unimodal test items. Overall, this pattern of results is more in line with a study-test overlap explanation than a multisensory benefit explanation.

In our current experiments participants had to remember both sounds and pictures in the same series of stimuli so that both modalities were task-relevant. We speculated that the multisensory memory benefit might be stronger if both modalities were task-relevant. In our previous study (Pecher & Zeelenberg, 2022) only one modality was task-relevant, and we obtained no multisensory benefit in contrast to previous studies (Heikkila et al., 2015, 2017b; Thelen et al., 2015). In previous studies, task instructions were to remember stimuli from one modality, but because participants performed both picture memory and sound memory tasks, they may have unintentionally treated both modalities as task-relevant. To increase the likelihood of finding effects of multisensory study, we used a task in which both modalities were task-relevant. Nevertheless, we did not obtain evidence that multisensory processing increased memory performance.

The results of Experiment 1 showed that memory was not better when picture and sound were presented simultaneously during study than when they were separated by other study items, as was also observed by Meyerhoff and Huff (2016) in memory for video clips. The finding that temporal overlap did not result in a memory benefit is problematic for the multisensory integration explanation (Shams & Seitz, 2008). For multisensory integration to occur the sound and picture must be presented together so that they are integrated into one memory representation (Doehrmann & Naumer, 2008; Quak et al., 2015). One possible explanation for the lack of a multisensory memory benefit in the present study is that multisensory integration did not occur in the bimodal condition. In our view, such an explanation, however, is neither attractive nor plausible. It is not attractive because it involves a risk of circularity. If a multisensory memory benefit is found it is explained by multisensory integration. If no multisensory memory benefit is found then it is due to a lack of multisensory integration. Moreover, we deem such an explanation implausible because neuro-imaging studies have indicated a network of temporal and frontal regions that are involved when congruent multisensory stimuli are processed (Doehrmann & Naumer, 2008), suggesting that sensory integration readily occurs, at least for congruent stimuli (Quak et al., 2015). Our results suggest, however, that such sensory integrative processing does not result in a memory benefit. Rather, the finding that memory performance was at least as good when sound and picture were presented on separate trials during study suggests that memory was strengthened at the conceptual level rather than at the sensory level (see also Heikkilä et al., 2017b). A sound and a picture of the same item, for example of a dog, have no sensory features in common, so when they are presented on separate trials the only link is the concept *dog*. A possible mechanism to explain the memory advantage for the items that were presented on separate study trials is that on both trials the concept was activated, resulting in better memory performance for the concept after presentation in two modalities than after one presentation in one modality. Based on the results from Experiment 1, one might speculate that encoding variability resulted in better memory performance (Glenberg, 1979; Maddox, 2016; Verkoeijen et al., 2004), but the results of Experiment 2 show that repetitions in the same modality resulted in even better memory performance. Thus, repetition in a different modality does increase memory performance somewhat, but not as much as repetition in the same modality, given that the study format is the same as the test format.

In Experiment 2 we found better performance for the bimodal study condition compared to the unimodal-both study condition. Here, however, the results are also better explained by study-test overlap. In the bimodal condition, the picture and sound are both presented on the two study presentations. Thus, both picture and sound were studied twice. In the unimodal-both condition, the picture and sound were each studied once. Thus, in all test conditions, the relevant stimulus format was studied twice in the bimodal condition, and once in the unimodal-both condition. Therefore, the advantage of bimodal study over unimodal-both study shows a benefit of repeating the item in the format in which it is tested. Note that in the unimodal test conditions (i.e., sound only and picture only), we found no benefit of having presented the other modality during study, as performance was not better in the bimodal study condition than in the unimodal study condition. In fact, memory performance for items tested unimodally (i.e., picture only or sound only) was better for items presented in the same unimodal format as during study than for items studied in a bimodal format. This finding is inconsistent with the idea that multisensory integration causes a memory benefit.

Although our findings contrast with some previous studies that have obtained a multisensory study advantage for items tested in one modality (Duarte et al., 2023; Lehmann & Murray, 2005; Moran et al., 2013; Thelen et al., 2015; but see Cohen et al., 2009; Nyberg et al., 2000), they are in line with studies showing benefits of study-test format overlap (Meyerhoff et al., 2022; Pecher & Zeelenberg, 2022). This is consistent with the encoding specificity and transfer-appropriate processing principles (Blaxton, 1989; Cabeza, 1994; Morris et al., 1977; Parks, 2013; Roediger & Blaxton, 1987; Roediger et al., 1989; Tulving & Thomson, 1973; Zeelenberg, 2005) which predict that memory performance will be better with greater overlap between processing during study and test. Interestingly, the Intersensory Redundancy Theory (Bahrick & Lickliter, 2014) states that participants will pay more attention to amodal features and less to modality-specific features for multisensory items than for unisensory items. Although this theory focusses on attention, we can speculate that memory performance will be better if attention at study and test are focused on the same features than if attention is focused on different features (Barclay et al., 1974; Zeelenberg et al., 2003). Thus, these views predict that a multisensory benefit will occur when items at test are also multisensory but not when they are unisensory at test.

To conclude, our results indicate that the benefit of multisensory study on memory is not due to multisensory integration at study. Rather, in some studies the benefit might have been due to the additional information that is provided in multimodal presentations compared to unimodal presentations. Even though both the visual and auditory modalities were task-relevant in our experiments, we did not replicate the multisensory benefit and instead argue that memory performance benefits most from the overlap between study and test format.

## Data Availability

All data that were used for the analyses, figures, and tables are available via the Open Science Framewor at: https://osf.io/9m2jv/

## Appendix A

### Experimental Items

#### Experiment 1

*Accordion, aerosol can, alarm, alarm clock, ambulance, apple, baby, balloon, bell, billiards, book, bowling pin, cup, broom, phone, camel, camera, canary, cannon, car, cash register, castanets, cat, chain, chainsaw, chalkboard, champagne, chicken, chisel, choir, church, hands, coin, copier, cow, cricket, dice, didgeridoo, dog, dolphin, donkey, water tap, drums, duck, eagle, electric saw, elephant, fireworks, flute, fly, frog, frying pan, gavel, ghost, goat, gong, guillotine, guinea pig, guitar, gun, hair dryer, hammer, harmonica, harp, helicopter, horse, hyena, jetplane, keyboard, knife, lamb, latex gloves, light saber, lips, padlock, microwave, monkey, mosquito, computer mouse, nose, owl, peacock, pencil, penguin, piano, pig, pill bottle, pingpong bat, plunger, potato chips, pitcher, race car, railroad crossing, rain, raven, robot, rooster, rubber duck, saw, saxophone, school bell, scissors, seagull, sewing machine, shaver, sheep, shower, sleigh bells, slot machine, soda can, leopard, stapler, submarine, swan, sword, telephone, toaster, toilet, torch, paper, truck, trumpet, typewriter, vacuum cleaner, violin, whale, whistle, witch, wolf, zipper.*

#### Experiment 2

*Accordion, aerosol can, alarm clock, alarm, ambulance, anchor, apple, axe, baby, balloon, banjo, baseball mitt, bat, bath, bell, billiards, biplane, boiling water, book, boot spurs, bowling pin, boxing gloves, broom, brush, phone, camel, camera, campfire, canary, cannon, car, cash register, castanets, cat, chain, chainsaw, chalkboard, cheering audience, chicken, chisel, choir, church, hands, clock, clown, coffeemaker, coin, copier, cow, cricket, crocodile, dice, didgeridoo, dinosaur, dog, dolphin, donkey, door, drill, water tap, drums, duck, dynamite, eagle, electric saw, elephant, elevator, fan, fire extinguisher, fire truck, fireworks, flag, flute, fly, football, fountain, french horn, frog, frying pan, gavel, ghost, goat, golf ball, gong, gorilla, grenade, grizzly bear, guillotine, guinea pig, guitar, gun, hair dryer, hammer, harmonica, harp, heart monitor, helicopter, hockey stick and puck, horse, jetplane, kettle, key, keyboard, kitchen stove, knife, plank, lamb, latex gloves, lawn mower, leopard, lighter, light saber, light switch, lion, lips, padlock, blood pressure device, maracas, metronome, microwave, mobile phone, monkey, mosquito, motorboat, motorcycle, mouse, computer mouse, nail clippers, nose, owl, parrot, peacock, pencil, pencil sharpener, penguin, piano, pill bottle, pingpong bat, plunger, police car, potato chips, pitcher, printer, raccoon, race car, radio, railroad crossing, rain, rake, raven, record player, rhino, rifle, robot, rocket, rooster, rubber duck, salt shaker, saw, saxophone, school bell, scissors, seagull, sea lion, sewing machine, shaver, sheep, shoe, shower, sleigh bells, slot machine, soda can, songbird, stapler, submarine, swan, sword, telephone, toaster, toilet, toothbrush, torch, tornado, paper, toy ball, train, triangle, truck, trumpet, typewriter, vacuum cleaner, violin, water cooler, whale, whistle, witch, wolf, xylophone, zebra, zipper.*

## Footnotes

1 Due to a programming error, for the first 16 participants that we tested the ISI was 20 ms shorter and varied between 2,880 and 3,480 ms instead of 2,900 and 3,500 ms. Because all manipulations were within subjects and the difference was small, we deemed it unlikely that this affected the pattern of results.

2 The Cauchy scale was 0.707.

## Declarations

### Open practices statements

The data for all experiments are available via the Open Science Framework at: https://osf.io/9m2jv/ and no experiment was preregistered.
